# Study on the Characteristics of Coarse Feeding Tolerance of Ding’an Pigs: Phenotypic and Candidate Genes Identification

**DOI:** 10.3390/genes15050599

**Published:** 2024-05-08

**Authors:** Yanxia Song, Mingming Xue, Feng Wang, Qiguo Tang, Yabiao Luo, Meili Zheng, Yubei Wang, Pengxiang Xue, Ningqi Dong, Ruiping Sun, Meiying Fang

**Affiliations:** 1 Sanya Institute of China Agricultural University, Sanya 572024, China; songyanxia2023@126.com (Y.S.); pq007007@126.com (Y.W.); shiyiyyh@163.com (N.D.); 2 Department of Animal Genetics and Breeding, National Engineering Laboratory for Animal Breeding, MOA Key Laboratory of Animal Genetics and Breeding, Beijing Key Laboratory for Animal Genetic Improvement, State Key Laboratory of Animal Biotech Breeding, Frontiers Science Center for Molecular Design Breeding, College of Animal Science and Technology, China Agricultural University, Beijing 100193, China; mingmxue@163.com (M.X.); tango@cau.edu.cn (Q.T.); yabiaoluo2021@163.com (Y.L.); xuepengxiang98@163.com (P.X.); 3 Institute of Animal Science and Veterinary Medicine, Hainan Academy of Agricultural Science, Haikou 571100, China; wfeng73cn@sina.com (F.W.); ruiping937@126.com (R.S.); 4 Beijing General Station of Animal Husbandry, Beijing 100107, China; 15652827528@163.com

**Keywords:** Ding’an pigs, Hainan pigs, coarse feeding tolerance, crude fiber, transcriptome sequencing, WGCNA

## Abstract

Ding’an (DA) pig, a prominent local breed in Hainan Province, exhibits notable advantages in coarse feeding tolerance and high-quality meat. To explore the potential genetic mechanism of coarse feeding tolerance in DA pigs, 60-day-old full sibling pairs of DA and DLY (Duroc-Landrace-Yorkshire) pigs were subjected to fed normal (5%) and high (10%) crude fiber diets for 56 days, respectively. The findings showed that increasing the crude fiber level had no impact on the apparent digestibility of crude fiber, intramuscular fat, and marbling scores in DA pigs, whereas these factors were significantly reduced in DLY pigs (*p* < 0.05). Through differential expression analysis and Weighted Gene Co-expression Network Analysis (WGCNA) of the colonic mucosal transcriptome data, 65 and 482 candidate genes with coarse feeding tolerance in DA pigs were identified, respectively. Joint analysis screened four key candidate genes, including *LDHB*, *MLC1*, *LSG1*, and *ESM1*, potentially serving as key regulated genes for coarse feeding tolerance. Functional analysis revealed that the most significant pathway enriched in differential genes associated with coarse feeding tolerance in Ding’an pigs was the signaling receptor binding. The results hold substantial significance for advancing our understanding of the genetic mechanisms governing coarse feeding tolerance in Ding’an pigs.

## 1. Introduction

The Ding’an pig, a well-established local breed in Hainan Province, is named for its predominant presence across the townships of Ding’an County. It constitutes one of the four major groups of Hainan pigs, alongside the Tun’chang, Lin’gao, and Wen’chang pig groups [1]. This breed is characterized by its small body size (approximate weight of 66 kg at seven months of age), black back and white belly, inverted eight-fold facial wrinkles, and a hanging upside-down white triangular star on the middle of the forehead [2]. The Ding’an pig is known for its advantageous properties, such as coarse feeding tolerance, tolerance to high temperature, tender meat quality, and lower susceptibility to diseases [3,4]. However, in recent years, Ding’an pigs have been affected by foreign pig breeds, African swine fever, and other factors, resulting in a continuous decline in their population and even endangering their survival. Moreover, the analysis of Ding’an pig characteristic traits is insufficient, which significantly hampers the development of the local pig industry in Hainan.

Coarse feeding tolerance refers to the ability to adapt to and digest feed ingredients with high fiber content [5,6]. This is described as the phenomenon where feeding rations with high fiber content have little impact on pig production traits [7]. Researchers typically measured pigs’ coarse feeding tolerance characteristics by assessing the apparent digestibility of crude fiber or determining the maximum crude fiber content in the feed for a constant daily gain and feed-to-gain ratio within the same pig breed. Alternatively, they might compare the daily weight gain and feed weight ratio among different pig breeds when fed high-fiber diets [8]. The primary breeds farmed in China are the modern cosmopolitan lean breeds, which are raised using expensive high-protein feeds. Feed expenditure constitutes over 70% of the total costs [9]. Compared to local Chinese pig breeds, these pigs exhibit limited utilization of roughage, leading to increased production expenses [10]. During periods of declining pork prices, farms often opted to augment the inclusion of roughage in feeds, replacing a portion of high-nutrient feed to mitigate costs. However, this practice could trigger a series of stress responses in pigs [11]. Increasing the fiber content in the feed could improve pig performance, reduce environmental pollution from pig manure, and make better use of fiber resources [12]. The coarse feeding tolerance of pigs is greatly influenced by pig breeds. Chinese local pig breeds are generally more resistant to roughage than commercial pig breeds [13]. Compared to “Duroc × Landrace × Yorkshire” pigs, fecal bacteria from Diqing Tibetan pigs showed significantly higher capability in breaking down neutral detergent fiber (NDF) (*p* < 0.05). In addition, there was no significant difference in the fiber degradation efficiency between the NDF 20% and NDF 40% groups of Diqing Tibetan pigs [14]. Pu et al. [7] found that increasing dietary fiber from 16.70% to 24.11% for Suhuai finishing barrows over a short period of 14 days did not impact average daily feed intake (ADFI) or feed conversion (F/G). The above studies on coarse feeding tolerance in pigs have mainly focused on phenotype collection. However, relatively few studies have been conducted on the regulatory mechanisms of pigs themselves.

Transcriptome sequencing technology has been used to study the genetic mechanism related to pork quality, reproduction, and other traits [15,16]. Lange et al. [17] conducted a study examining the impact of five distinct fiber types on the transcriptome of mouse colon mucosa. They discovered that PPAR-γ, a nuclear receptor, potentially acted as a critical upstream regulator, influencing the modulation of gene expression in colon mucosa induced by dietary fibers. Tang et al. [18]. studied the meat quality characteristics of 20 Ding’an pigs at 10 months of age and collected data on the longissimus dorsi muscle fatty acid and amino acid contents of Ding’an pigs. Currently, there is a lack of research on phenotypic data on coarse feeding tolerance characteristics and the identification of coarse feeding tolerance genes using transcriptome analysis in Ding’an pigs.

This study investigated the effects of feeding diets with 5% and 10% crude fiber on the production performance, carcass traits, meat quality, apparent crude fiber digestibility, and serum biochemical parameters of 60-day-old Ding’an and Duroc-Landrace-Yorkshire pigs. Subsequently, the colonic mucosa transcriptome of pigs was sequenced to identify key genes and signaling pathways associated with crude fiber tolerance. This study will provide a basis for studying the molecular regulation mechanism of crude fiber tolerance in Ding’an pigs.

## 2. Materials and Methods

### 2.1. Experimental Animal Feeding Management and Sample Collection

All animal husbandry and sample collection processes were in accordance with the ethical regulations of the State Key Laboratory of Agricultural Biotechnology of the Animal Welfare Committee of China Agricultural University. The experimental pigs were provided by Hainan Qingping Agricultural Products Development Co., Ltd. (Ding’an, China), and the experiment was completed at Yongfa Research and Experimental Base of Animal Husbandry and Veterinary Research Institute of Hainan Academy of Agricultural Sciences. Eight 60-day-old full-sibling Duroc-Landrace-Yorkshire (DLY) piglets were divided into 2 groups, with 4 piglets (4 sows, 4 litters) in each group. The groups were designated as the high-fiber diet (10% crude fiber) group (DLYH) and the normal-fiber diet (5% crude fiber) group (DLYN). Similarly, eight 60-day-old Ding’an (DA) pigs were also assigned to 2 groups, with 4 pigs (2 castrate boars and 2 sows, 2 litters) in each group. The groups were labeled as the high-fiber diet (10% crude fiber) group (DAH) and the normal-fiber diet (5% crude fiber) group (DAN). The initial weights of pigs in DAH, DAN, DLYH, and DLYN groups were 10.62 ± 1.19 kg,10.65 ± 0.53 kg, 14.83 ± 1.96 kg, 15.00 ± 0.84 kg, respectively. DA pigs and DLY pigs were weaned at 25 days of age. The pigs were fed a commercial diet containing 5% crude fiber under the same feeding conditions before the experiment. The experimental diets were formulated according to the NRC (2020) Nutritional Requirements of Swine, and the composition and nutrition details of the diets can be found in Supplementary Table S1, which quoted from Pengxiang Xue’s article [19]. Each group was housed in a single pen throughout the experimental period, and each pig was individually housed in a separate pen during manure collection three days before the end of the experiment. Throughout the 56-day trial period, all pigs were raised and managed under the same conditions, with adequate access to feed and water. At the end of the experiment, all pigs were euthanized, and experimental samples and phenotypic data were collected. The samples of intestinal mucosa were scraped with sterile slides and stored in tubes. All samples were stored at −80 °C using liquid nitrogen quick-freezing.

### 2.2. Collection of Phenotypes

#### 2.2.1. Measurement of Growth Performance

Weighing and recording the body weight of each of the sixteen pigs at the start and finish of the trial, respectively. Recording the daily feed intake for each group. Average daily gain (ADG, kg/d; (Final weight per pig − initial weight per pig)/56), average daily feed intake (ADFI, kg/d; Feed intake/56), and feed-to-weight ratio (F/G; ADFI/ADG) were calculated.

#### 2.2.2. Measurement of Apparent Digestibility of Crude Fiber

The apparent digestibility of CF was determined by the total feces collection method [20]. During the final 72 h of the experiment, the intake of dietary crude fiber was recorded for each of the sixteen pigs, and all fecal samples from each of the sixteen pigs were collected. The total fecal samples for each of the sixteen pigs were weighed and thoroughly mixed. 20% of the total fecal samples were placed in a 65 °C oven for 72 h to determine the initial moisture content. After rehydration for 24 h to constant weight, the samples were finely ground using a 40-mesh sieve and packaged for testing. The determination of crude fiber content was conducted in accordance with the standard GB/T 6434-2022 [21].

The calculation method for the apparent digestibility of crude fiber was as follows:

Apparent digestibility of crude fiber (%) = (Amount of crude fiber intake − Amount of crude fiber in feces)/Amount of crude fiber intake × 100%.

#### 2.2.3. Measurement of Carcass Traits

The measurement of carcass traits was referred to against “Technical Specification for Measurement of Carcass Traits of Lean Pigs” (NY/T 825-2004) [22]. At the end of the experiment, the dressing rate (%), backfat thickness (cm), and loin eye area (cm^2^) of each of the sixteen pigs were measured. The dressing rate was calculated as the ratio of carcass weight to live weight. Backfat thickness was assessed by measuring the subcutaneous fat thickness between the 6th and 7th ribs using a vernier caliper. Loin eye area = Height of the loin muscle × Width of the loin muscle × 0.7 [23].

#### 2.2.4. Measurement of Meat Quality Traits

All of the 16 pigs were slaughtered at the end of the trial, and meat quality traits in pork were assessed in compliance with the “Technical Regulations for Pork Quality Determination” (NY/T 821-2019) [24], which measured various indices such as marbling score, pH_45 min_, pH_24 h_, shear force (N), drip loss (%), and intramuscular fat (g/100 g). The longest dorsal muscle was sampled from the 10th to 12th ribs to determine each meat quality trait index. Marbling Score was determined according to the National Pork Producers Council (NPPC) standards. The pH of the longest muscle of the back of the pig after slaughter was determined by a pH meter (Testo SE & Co KGaA, Neustadt, Germany) at 4 °C for 45 min and 24 h, respectively.

Drip loss was assessed by suspending approximately 100 g of the longest dorsal muscle (5 cm × 3 cm × 2 cm, 3 technical replicates) at 4 °C for 24 h. Drip loss was calculated based on the amount of water lost [25]. We sampled 400 g of the longest back muscle, and the meat samples were allowed to mature. Once the temperature at the center of the muscle reached 70 °C, the heating process was halted, and the meat samples were removed and cooled to 20 °C. Ten pieces of meat were cut using the Tenderness Tester (CLM3B, Beijing Tianxiang Feiyu Instrument Co., Ltd., Beijing, China), each measuring 5 cm × 1 cm × 1 cm, perpendicular to the muscle fibers. The shear force was then measured, with a total of 10 technical replicates for each sample and averaged, The samples of the longest back muscle were frozen and preserved. The intramuscular fat content was determined using the Soxhlet extraction method in accordance with the National Standard for Food Safety for the Determination of Fat in Food (GB 5009.6-2016) [26].

#### 2.2.5. Measurement of Serum Biochemical Indicators

During the slaughter experiment, sixteen blood samples were collected and then centrifuged to separate the serum. Commercial kits were employed to determine the levels of total protein (TP, g/L), albumin (ALB, g/L), alanine transaminase (ALT, U/L), aspartate aminotransferase (AST, U/L), total cholesterol (TC, mmol/L), triglycerides (TG, mmol/L), and blood glucose (GLU, mmol/L) in the serum.

### 2.3. Total RNA Extraction and Transcriptome Sequencing

The extraction of total RNA from 16 colonic mucosa tissues was performed using the TRIzol method. RNA was tested for contamination and degradation using 1% agarose gel electrophoresis. RNA purity and concentration were detected by Nanodrop (Thermo Scientific NC2000, Wilmington, DE, USA). The integrity of RNA fragments was assessed using the Agilent 2100 Bioanalyzer (Agilent Technologies, Santa Clara, CA, USA). 

After the samples passed the test, single-stranded cDNA was synthesized through reverse transcription, utilizing six-base random primers and the fragmented mRNA as a template. Subsequently, double-stranded cDNA was synthesized by adding buffer, dNTPs, and DNA polymerase I. Sixteen cDNA libraries were constructed via a series of steps, including purification of double-stranded cDNA, end repair, A-tailing, adapter ligation, and PCR amplification. Initially, the size of insert fragments within the libraries was assessed using Agilent 2100 Bioanalyzer (Agilent Technologies, Santa Clara, CA, USA). Upon meeting the expected size, the effective concentration of the libraries (>4 nM) was precisely quantified via Q-PCR to ensure compliance with high-quality standards. 

Sixteen cDNA libraries were paired-end sequenced using the Illumina high-throughput sequencing platform (NovaSeq 6000, San Diego, CA, USA). The read length was 150 bp. The Sequencing was performed by Nanjing Allwegene Science and Technology Co., Ltd. (Nanjing, China). The raw reads underwent quality control screening using Trimmomatic software (version 0.39). Clean reads were obtained through a series of preprocessing steps, including the removal of adapters from raw data reads, exclusion of reads containing over 10% unidentifiable base information (N bases), and Q30 > 85%, indicating good sequencing quality. The Sus Scrofa 11.1 reference genome and relevant files were acquired from the Ensemble website (https://asia.ensembl.org/index.html, accessed on 21 May 2022). Following this, the clean reads were aligned to the porcine reference genome (Sus scrofa 11.1) utilizing HISAT2 software (version 2.2.1). This process ensured accurate mapping of the sequencing data to the reference genome for subsequent analysis.

### 2.4. Principal Component Analysis

Based on the genes identified in this study, PCA of all genes among colonic mucosal samples from the DAH, DAN, DLYH, and DLYN groups was performed using the R package to determine intra-individual group reproducibility and inter-group variability.

### 2.5. Differential Gene Analysis and Validation of Transcriptome Data Accuracy

The DESeq2 software (version 3.6.3) was utilized to detect significantly differentially expressed genes (DEGs), applying the criteria of fold change cut-off of >1.5 and false discovery rate (FDR) < 0.05. The Multiple test correction used the BH (Benjamini/Hochberg) method. Real-time fluorescence quantitative verification was used to verify the reliability of transcriptome data. The same tissue samples used for transcriptome sequencing were utilized for RNA extraction. Subsequently, 2 μg of RNA was reverse transcribed into cDNA using the Takara PrimeScript™ II 1st Strand cDNA Synthesis Kit. Eight DEGs were randomly selected from the colonic mucosa of the DAH vs. DLYH group, with *GAPDH* serving as the reference gene [27]. Primers were designed using Premier 5.0 software (Supplementary Table S2). Quantitative real-time PCR analysis was performed with the SYBR Fast Universal qPCR Kit (FOR SCIENCE). The relative expression of the genes was calculated using the 2^−ΔΔCt^ method.

### 2.6. Functional Analysis of DEGs

GO (Gene ontology, http://geneontology.org/, accessed on 3 December 2023) and KEGG (Kyoto Encyclopedia of Genes and Genomes, https://www.kegg.jp/kegg/ko.html, accessed on 5 December 2023) enrichment analysis of DEGs (Sixty-five candidate genes for coarse feeding tolerance in Ding’an pigs were identified through differential expression analysis) was conducted using the enrichplot and the clusterProfiler R package. The thresholds for significant GO terms and enriched KEGG pathways selection were set at false discovery rate (FDR) < 0.05.

### 2.7. Weighted Gene Co-Expression Network Analysis

The R package WGCNA (version 1.69) was used to analyze the gene expression data and construct a weighted gene co-expression network of differential mRNAs for all samples [28]. Briefly, the final expression matrix of 5000 probes was used to build the co-expression network. A soft threshold of 13, determined based on the sft$powerEstimate return value, was applied to create an unsigned topological overlap matrix (Supplementary Figure S1). Hierarchical clustering was then conducted to ensure a minimum of 30 genes within each module, with a threshold of 0.25 set for merging similar modules. After dividing the genes into different modules, the eigenvalues of the modules were calculated, and the correlation between the modules and the differential phenotype values was analyzed. We calculated gene significance (GS), module significance (MS), and module membership (MM). GS and MS were used to identify the modules most relevant to the target phenotype. Hub genes within a selected module were identified based on both GS and MM, indicating high relevance to the target trait.

### 2.8. Network of Differential Gene-Protein Interactions

We conducted protein-protein interaction (PPI) network analyses using the Sus Scrofa STRING database for Ding’an pig coarse feeding tolerance candidate genes identified through differential expression analysis and WGCNA. The resulting data were then visualized and analyzed in Cytoscape 3.9.1 software, enabling a comprehensive exploration of the molecular interactions and functional relationships among the identified genes. 

### 2.9. Statistical Analysis of Data

The general linear model (GLM) in SPSS 26.0 was utilized for two-way ANOVA to analyze differences in phenotypic data between pig breeds and crude fiber levels. Duncan’s method was used for multiple comparisons, and the results were expressed as mean ± standard error, with *p* < 0.05 indicating significant differences. And GraphPad Prism 9 was used for graphing.

## 3. Results

### 3.1. Differential Phenotypic Collection between DA (Ding’an) Pigs and DLY (Duroc-Landrace-Yorkshire) Pigs

**Figure 1:**
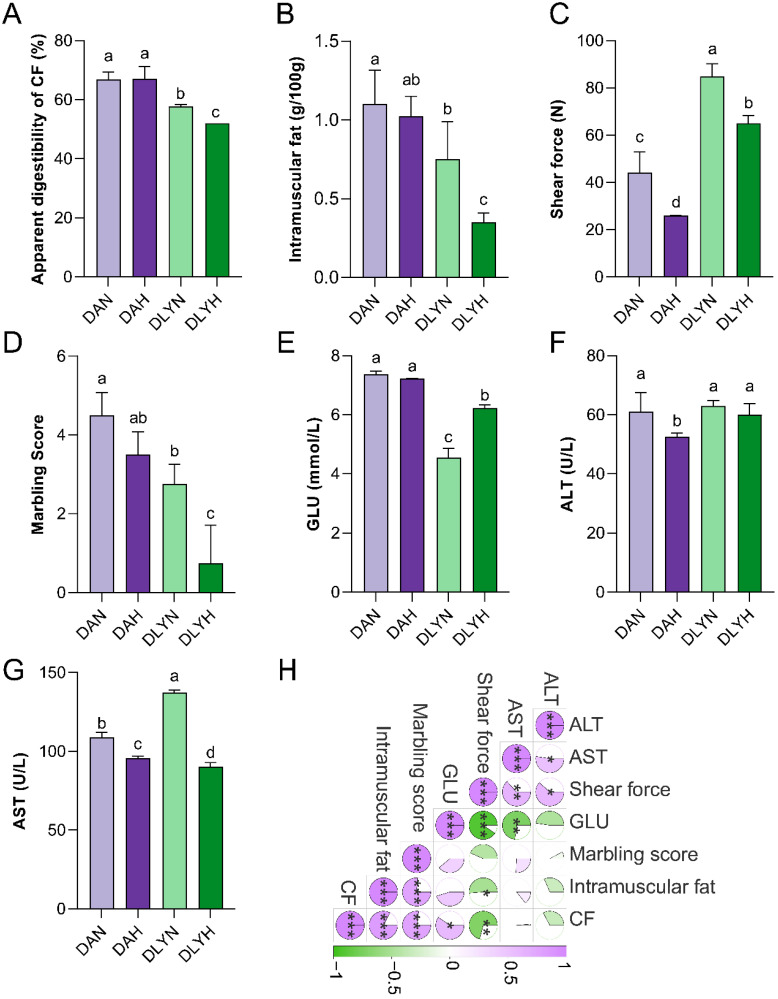
Effect of pig breed and crude fiber level on phenotype indicators. (**A**) Apparent digestibility of CF. (**B**) Intramuscular fat. (**C**) Shear force. (**D**) Marbling score. (**E**–**G**) GLU, ALT, AST. (**H**) Pearson correlation analysis of indicators. DAN: normal (5%) crude fiber ration group for Ding’an Pigs; DAH: high (10%) crude fiber ration group for Ding’an Pigs; DLYN: normal (5%) crude fiber ration group for DLY Pigs; DLYH: high (10%) crude fiber ration group for DLY Pigs. ^a–d^ Values within one and the same measurement indicator with different superscripts differ significantly (*p* < 0.05). Different lowercase letters indicated significant difference (*p* < 0.05); * *p* < 0.05, ** *p* < 0.01, *** *p* < 0.001. The data on the apparent digestibility of CF were partly quoted from Pengxiang Xue’s article [19].

Compared to DLY pigs, DA pigs had higher apparent digestibility of CF, intramuscular fat content, marbling score, and lower shear force value in DA pigs (Figure 1A–D). This indicated that DA pigs exhibited higher digestibility of crude fiber and better meat quality. When increasing the crude fiber level within the same pig breed, the difference in apparent digestibility of CF, intramuscular fat content, and marbling score between the DAH and DAN groups was not significant. However, the DLYH group exhibited significantly lower apparent digestibility of CF, intramuscular fat, and marbling scores than the DLYN group (Figure 1A,B,D) (*p* < 0.05). Moreover, increasing the crude fiber level significantly reduced the shear force in both DA and DLY pigs (*p* < 0.05), with the shear force value in the DAH group being lower than that in the DLYH group (Figure 1C) (*p* < 0.05). These results indicated that the apparent digestibility of CF was not affected in DA pigs but decreased in DLY pigs after increasing the crude fiber level (Figure 1A). Increasing the crude fiber level significantly improved meat tenderness in both DA and DLY pigs, with a greater improvement observed in DA pigs. Additionally, marbling score and intramuscular fat content were not affected in DA pigs but were significantly reduced in DLY pigs (Figure 1B,D).

Compared with DLY pigs, The GLU level of DA pigs significantly increased (*p* < 0.05). After increasing the crude fiber level of the same pig breed, the ALT and AST levels in the DAH group were significantly lower than those in the DAN group (*p* < 0.05). The AST of the DLYH group was significantly lower than that of the DLYN group (*p* < 0.05), indicating that increasing crude fiber levels could protect the liver function of pigs to a certain extent. The difference in GLU between DA pigs was not significant, but DLY pigs showed a significant increase in GLU (*p* < 0.05), indicating that increasing crude fiber enhanced the metabolic capacity of DLY pigs (Figure 1E–G).

**Figure 2:**
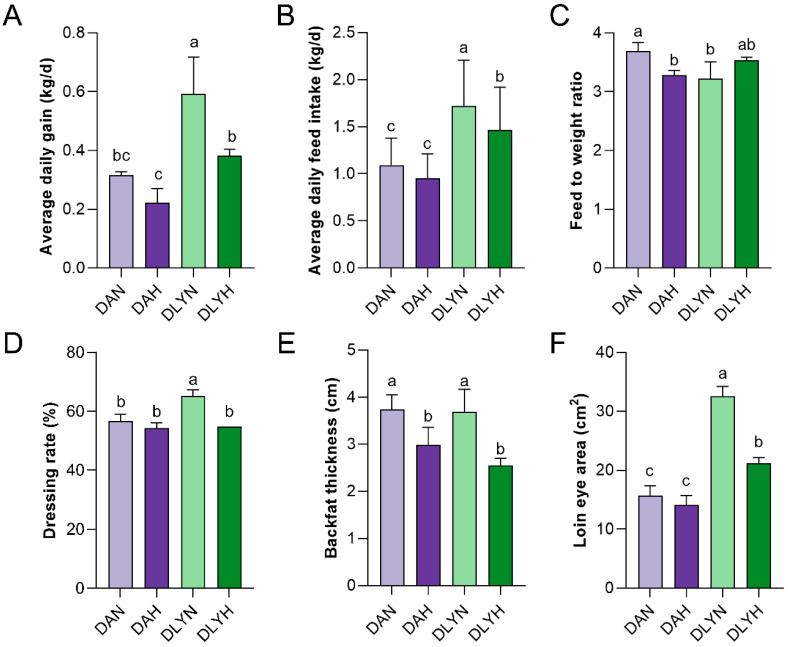
Effect of pig breed and crude fiber level on Indicators. (**A**) Average daily gain (kg/day). (**B**) Average daily feed intake (kg/day). (**C**) Feed to weight ratio. (**D**) Dressing rate (%). (**E**) Backfat thickness (cm). (**F**) Loin muscle area (cm^2^). ^a–d^ Values within one and the same measurement indicator with different superscripts differ significantly (*p* < 0.05). The data on the feed-to-weight ratio and dressing rate were partly quoted from Pengxiang Xue’s article [19].

In comparison to DLY pigs, DA pigs had significantly lower average daily weight gain, average daily feed intake, and loin eye area (*p* < 0.05). This suggested that the growth performance and carcass traits of DA pigs were lower. After increasing the crude fiber level within the same pig breed, the backfat thickness of both DA and DLY pigs significantly decreased (*p* < 0.05). There was no significant difference in average daily weight gain, average daily feed intake, dressing rate, and loin eye area between the DAH and DAN groups. However, the average daily weight gain, average daily feed intake, slaughter rate, and loin eye area of the DLYH group were significantly lower than those of the DLYN group (*p* < 0.05); the feed-to-weight ratio of the DAH group was significantly lower than that of the DAN group (*p* < 0.05). It indicated that increasing the level of crude fiber did not affect the average daily weight gain, average daily feed intake, dressing rate, and loin eye area of DA pigs, while DLY pigs showed a significant decrease (Figure 2). There were no significant differences in PH_24 h_, PH_45 min_, drip loss, ALB, TC, TG, and TP among the four groups (Supplementary Figures S2 and S3).

### 3.2. Inter-Phenotypic Correlation Analysis

According to inter-phenotypic correlation analysis (Figure 1H), the apparent digestibility of CF showed a significant positive correlation with intramuscular fat, marbling score, and GLU (*p* < 0.05) and a significant negative correlation with shear force (*p* < 0.05). Intramuscular fat showed a significant positive correlation with marbling score (*p* < 0.05) and a significant negative correlation with shear force (*p* < 0.05), GLU was negatively correlated with shear force and AST (*p* < 0.05), Shear force showed a significant positive correlation with AST and ALT (*p* < 0.05), AST showed a significant positive correlation with ALT (*p* < 0.05). In addition, the correlation between GLU and shear force was over 0.8.

### 3.3. Analysis and Validation of Differentially Expressed Genes (DEGs)

**Figure 3:**
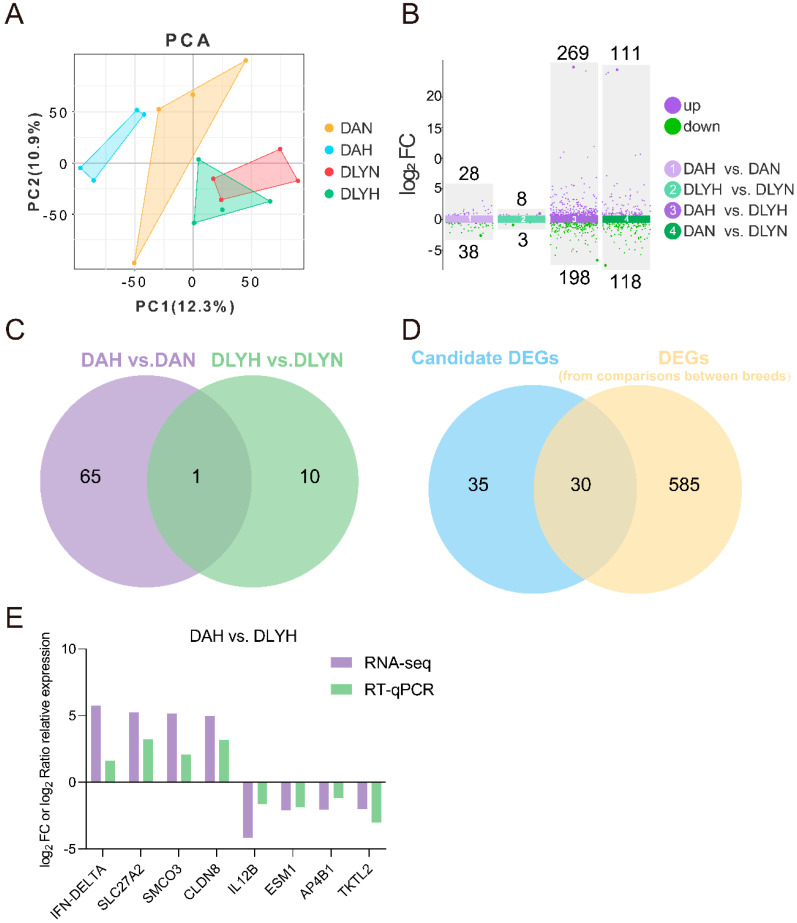
(**A**) PCA of all samples. (**B**) Differential genes in the four comparison groups. (**C**,**D**) Venn diagrams for differential genes. (**E**) Validation of the correlation of RT-qPCR with RNA-seq data for DEGs in DAH vs. DLYH group, *x*-axis was gene name, *y*-axis was log_2_ FC or log_2_ Ratio relative expression.

PCA showed that the samples within the DAH, DAN, DLYH, and DLYN groups clustered together well, while intergroup samples of four groups showed separation (Figure 3A), indicating that the data in this study were reliable and had good intragroup consistency and intergroup variability. In the DAH vs. DAN, DLYH vs. DLYN, and DAH vs. DLYH and DAN vs. DLYN comparator groups, 66, 11, 467, and 229 DEGs were respectively screened. Among these, 28 DEGs in the DAH group, 8 in the DLYH group, 269 in the DAH group, and 111 in the DAN group were up-regulated, while 38, 3, 198, and 118 were respectively down-regulated (Figure 3B, Supplementary Table S3). Eight DEGs were randomly selected from the DAH vs. DLYH group, and the accuracy of the RNA-Seq data was verified by RT-qPCR (Figure 3E). The expression patterns of these eight genes were found to be consistent with the RNA-Seq results, confirming the reliability of the sequencing data.

The Venn analysis of DEGs in the DAH vs. DAN and DLYH vs. DLYN groups was conducted (Figure 3C). One gene existed in both comparative groups, while 65 genes were exclusively differential expressed in the DAH vs. DAN group. These 65 genes (Supplementary Table S4) might be candidate genes for coarse feeding tolerance differences in DA pigs. Furthermore, we merged the number of DEGs between the two pig breeds at the same diet level (DAH vs. DLYH and DAN vs. DLYN) to 615. Subsequently, the 615 DEGs from comparisons between pig breeds were subjected to Venn analysis with the initial 65 candidate DEGs. The analysis revealed that 30 DEGs were shared, indicating they may be genes involved in differences in traits, including coarse feeding tolerance trait, between the two pig breeds (Figure 3D).

### 3.4. Functional Enrichment Analysis of Candidate DEGs for Coarse Feeding Tolerance

**Figure 4:**
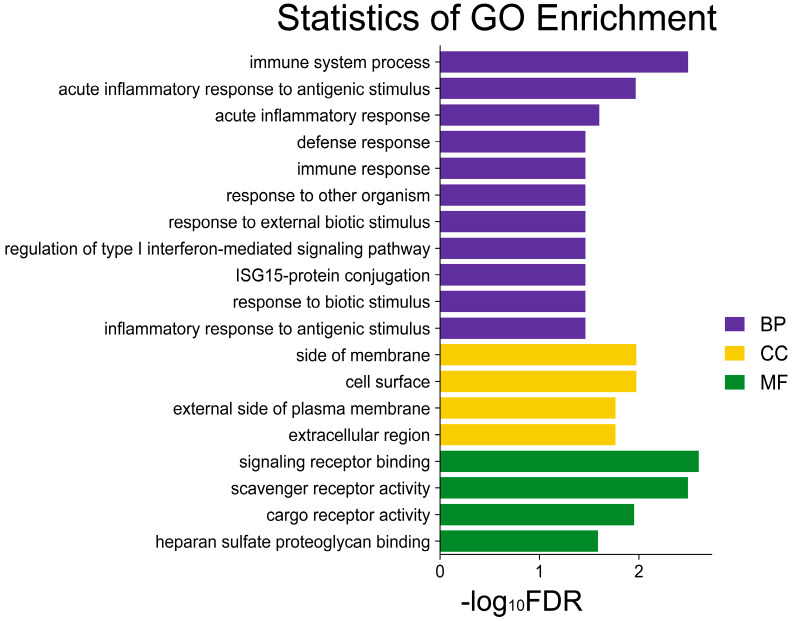
GO analysis of 65 candidate DEGs for coarse feeding tolerance in Ding’an pigs. BP, biological process; CC, cellular component; MF, molecular function.

GO analysis of the 65 DEGs yielded 19 significantly enriched GO terms (FDR < 0.05) (Figure 4). These included 11 biological process-related terms, such as immune system process, defense response, immune response, and other immune processes; four cellular component-related terms were the external side of the plasma membrane, extracellular region, etc., and four molecular function entries related to signaling receptor binding, scavenger receptor activity, and others.

KEGG pathway analysis of the 65 DEGs yielded 0 significant differential pathways (FDR < 0.05).

### 3.5. Weighted Gene Co-Expression Network Analysis (WGCNA) of All Expressed Genes 

**Figure 5:**
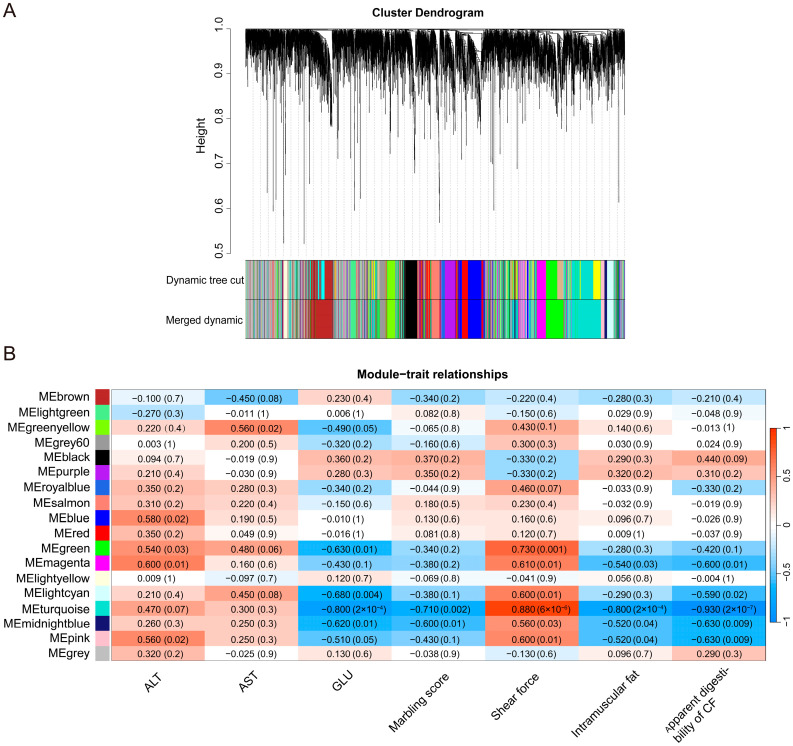
WGCNA of all genes in the colonic mucosa of four groups. (**A**) Hierarchical clustering tree diagram of 18 modules of co-expressed genes. Different colors represent different modules. (**B**) Module–trait relationship heatmap. Each row represented a module eigengene, and each column represented a phenotype trait.

WGCNA was performed to identify hub genes associated with coarse feeding tolerance traits in DA pigs. Cluster analysis was performed using TOM values, and 18 modules were constructed by partitioning the gene modules by hybrid dynamic shear (Figure 5A). 

The correlation of each module with coarse feeding tolerance phenotype in DA pigs is shown in Figure 5B. A single module showed highly significant module-trait correlations using |r| ≥ 0.7 and *p <* 0.01 as the determination criteria. Notably, the turquoise module showed a highly significant negative correlation with apparent digestibility of CF (r = −0.930, *p =* 2 × 10^−7^), GLU (r = −0.800, *p* = 2 × 10^−4^), intramuscular fat (r = −0.800, *p* = 2 × 10^−4^), and marbling score (r = −0.710, *p* = 0.002), and a highly significant positive correlation with shear force (r = 0.880, *p* = 6 × 10^−6^). 

Similarly, the analysis of gene significance across all modules showed a strong correlation between the turquoise module and the apparent digestibility of CF, GLU, intramuscular fat, marbling score, and shear force (Supplementary Figure S4). By drawing scatter plots of GS and MM, the turquoise module exhibited the closest association with the apparent digestibility of CF (cor = 0.89, *p* = 1 × 10^−200^) and shear force (cor = 0.83, *p* = 1 × 10^−200^) (Supplementary Figure S5). A total of 482 genes were filtered based on thresholds of |GS| < 0.6, |MM| < 0.6, and *p* < 0.05 (Supplementary Table S5). The GO and KEGG functional enrichment of all these genes in the turquoise module was shown in Supplementary Table S6.

### 3.6. Identification of Hub DEGs for Coarse Feeding Tolerance

**Figure 6:**
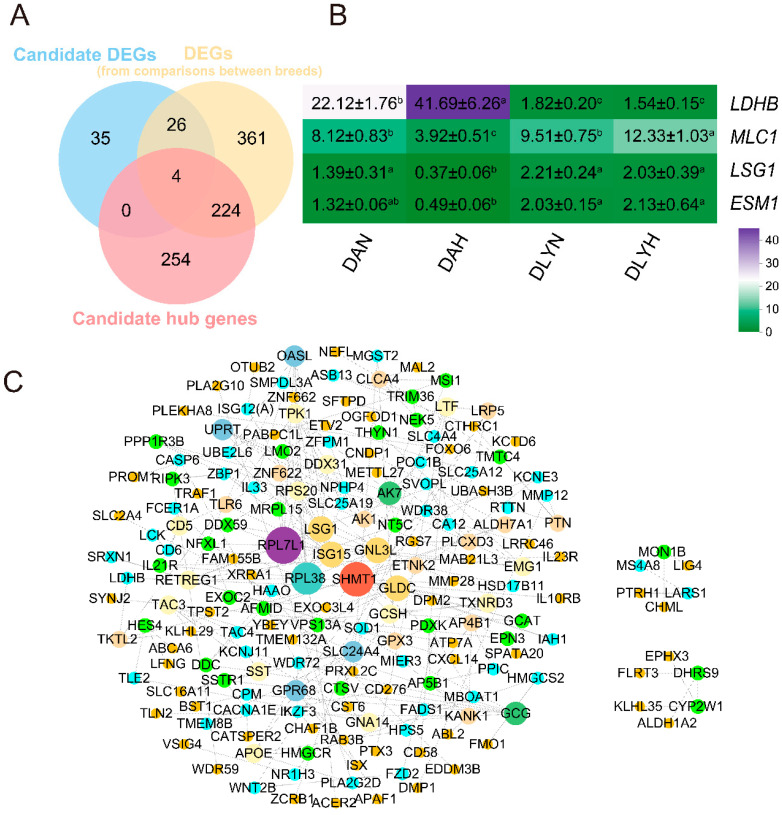
(**A**) Venn diagram of key candidate genes for coarse feeding tolerance in Ding’an pigs. (**B**) Heatmap of expression abundance (FPKM values) for four candidate genes from four groups. ^a–c^ Values within one and the same row with different superscripts differ significantly (*p* < 0.05). (**C**) Protein-protein interaction network diagram of candidate genes for coarse feeding tolerance in Ding’an pigs.

We performed a Venn diagram of 65 coarse feeding tolerance candidate genes, 615 breed-specific genes, and 482 genes in the turquoise module and identified four overlapping genes as key DA pig coarse-feeding tolerance candidate genes. Namely, *LDHB*, *MLC1*, *LSG1*, and *ESM1*. Among them, *LDHB* was key up-regulated genes in the DAH vs. DAN, and *MLC1* and *LSG1* were key down-regulated genes in the DAH vs. DAN. *ESM1* was a down-regulated gene in the DAH vs. DLYH (Figure 6A,B).

A total of 543 (65 + 482 merged) genes from candidate DEGs data and candidate hub gene data were used to perform PPI network analysis. In this network, *RPL7L1*, *SHMT1, ISG15*, *RPL38*, *LSG1*, *GNL3L*, and *GLDC* emerged as the central genes (Figure 6C). *ISG15* was involved in the signaling receptor binding, immune system process, extracellular region, defense response, immune response, response to other organisms, response to external biotic stimulus, regulation of type I interferon-mediated signaling pathway, and ISG15-protein conjugation. All of these pathways were coarse feeding tolerance candidate gene-enrichment pathways (Figure 4). 

## 4. Discussion

Research on coarse feeding tolerance traits is one of the hottest studies in livestock research. Ding’an pigs have coarse feeding tolerance traits [29], and there are fewer studies on their molecular mechanisms [30]. In this study, we constructed a database of coarse feeding tolerance phenotypes in DA pigs. Subsequently, a large number of genes related to coarse feeding tolerance in DA pigs were screened through transcriptome differential expression analysis and WGCNA analysis of the colon mucosa, which provided important clues for further research on regulatory mechanisms.

The coarse feeding tolerance characteristics of local pig breeds are generally measured by the apparent digestibility of crude fiber [31,32]. Generally, local pig breeds are more tolerant to roughage than foreign-introduced pig breeds. Du et al. [8] fed Two-flower face pigs and large white pigs with different bran total dietary fiber of 14.07%, 16.32%, 17.99%, and 18.85%, and found that the apparent digestibility of total dietary fiber (TDS) was notably higher in Two-flower face pigs compared to Large White pigs, and the fiber level exhibited no significant impact on the apparent digestibility of TDS in Two-flower face pigs. The apparent digestibility of CF was significantly higher in DA pigs than in DLY pigs in this study. When increasing the CF level, the apparent digestibility of CF was unaffected in DA pigs and decreased in DLY pigs, indicating that DA pigs were more tolerant of roughage feeding than DLY pigs.

The microbial degradation of crude fiber primarily occurs in the colon [33,34]. Previous studies have demonstrated that supplementation of pea and wheat bran crude fiber in pig diets resulted in an increase in *lactic acid* bacteria and *bifidobacteria* in colon contents, along with significant changes in gene expression related to mucosal immunity and barrier function [35,36,37]. These findings highlighted the importance of the colonic mucosa as the primary site for the absorption of decomposed crude fiber and suggested its significance in exploring the tolerance of DA pigs to coarse diets.

A total of 65 candidate DEGs associated with DA pig coarse feeding tolerance were identified through differential expression analysis and Venn analysis (Figure 2C). The Venn analysis was conducted on the 65 differential candidate genes and 615 comparative differential genes between the two pig breeds (DAH vs. DLYH and DAN vs. DLYN), revealing that 30 genes were co-expressed. Among them, 23 genes were expressed exclusively in DAN vs. DAH and DAH vs. DLYH, potentially indicating their role as candidate genes for comparative coarse feeding tolerance between the two breeds of pigs (Figure 6A). Twenty-three genes included *ANKRD40CL*, *CYP2W1*, *SLC51B*, *ISX*, *CA1*, *MMP28*, *CA12*, *ETNK2*, *UGDH*, *RASL12*, *CRYBG2*, *CPM*, *SLC4A4*, and *VPS13A*, which were up-regulated, while *CD6*, *TAP1*, and *SERPINB2* were down-regulated in both DAH vs. DAN and DAH vs. DLYH. It had been reported that the up-regulation of *SLC51B* expression inhibited the inflammatory response in the mouse colon, thus protecting against colonic mucosal injury, promoting bile acid transport, and maintaining the healthy homeostasis of the colonic mucosa [38,39]. The Intestine-specific homology cassette (*ISX)* is a homology cassette-containing protein belonging to a paired subfamily. This protein is phylogenetically homologous to *Pax3*, *Pax7*, and *Prrx1*. Studies involving targeted knockout of ISX in mice have shown that *ISX* was required for regulating the intestinal-specific expression of the high-density lipoprotein receptor, the cholesterol transport scavenger receptor type B, and vitamin A metabolism. This regulation contributed to maintaining healthy intestinal homeostasis [40]. The gene *CA12* encoded a zinc metalloenzyme known as carbonic anhydrase 12, which catalyzed the reversible reaction between carbon dioxide and bicarbonate. Its primary function is to act as a buffer, regulating intracellular pH in the intestinal tract [41,42]. *UGDH*, a cytoplasmic enzyme, catalyzes the oxidation of UDP-glucose to UDP-gluconate [43], participates in glycosaminoglycan and proteoglycan synthesis, and contributes to maintaining the integrity of the extracellular matrix [44,45]. Additionally, it was essential as a substrate for the production of hyaluronic acid and participates in drug and hormone metabolism through glucuronidation, consequently enhancing colonic immune responses [46,47,48]. *SLC4A4*, also known as electronegative sodium bicarbonate cotransporter protein isoform 1 (NBCe1), exhibited widespread expression in secretory and absorptive epithelia and played a vital role in regulating intracellular pH and facilitating transepithelial bicarbonate transport. It could modulate the microenvironment of the colon [49,50]. *CD6* is predominantly expressed by peripheral T lymphocytes, myeloid thymocytes, and B1a lymphocytes, constituting a type I transmembrane glycoprotein. It exerted an inhibitory effect on T-cell activation while also promoting colonic inflammatory responses [51,52]. Knockout of *TAP1* in mice led to the activation of their natural cell-killing capacity [53,54]. Collectively, these genes played crucial roles in maintaining the host immune response and intestinal health. Additionally, their up- and down-regulation may facilitate the adaptation of Ding’an pigs to high crude fiber level rations.

The 65 candidate DEGs primarily participate in immune responses, such as the immune system process, defense response, immune response, and so on. The most notable pathway identified in the GO functional enrichment analysis was the signaling receptor binding. The signaling receptor binding pathway exhibited 14 differential genes. These genes included *ESM1* (log_2_FC = −1.42), *TRAF1* (log_2_FC = −1.42), *ISG15* (log_2_FC = −1.34), *HPS5* (log_2_FC = −1.32), *TIGIT* (log_2_FC = −0.92), *LCK* (log_2_FC = −0.75), *TAP1* (log_2_FC = −0.74), *APOE* (log_2_FC = −0.66), and others, which are downregulated genes associated with coarse feeding tolerance in Ding’an pigs. *BMP3* (log_2_FC = 2.99), *GCG* (log_2_FC= 1.00), *SST* (log_2_FC = 0.88), *WNT2B* (log_2_FC= 0.69), and up-regulated genes linked to coarse feeding tolerance in Ding’an pigs. *LCK* was associated with the regulation of T-cell apoptosis [55]. *APOE*, known as apolipoprotein E, acts as a high-affinity ligand that promotes the clearance of various lipoproteins from the corpuscular circulation [56]. *APOE*-/- knockout germ-free (GF) mice fed either a fermentable fiber or a non-fermentable cellulose control diet were found to have reduced atherosclerosis in mice fed fermentable fiber [57]. *BMP3* promoted long-bone development in mice [58]. These candidate genes have been demonstrated to confer benefits to Ding’an pigs by promoting coarse feeding tolerance through the regulation of growth and enhancement of immune response.

In this study, we identified *RPL7L1*, *SHMT1, ISG15*, *RPL38*, *LSG1*, *GNL3L*, and *GLDC* hub genes by PPI network analysis of 543 (65 + 482 merged) coarse feeding tolerance candidate genes in Ding’an pigs. These hub genes participate in various immune pathways, including immune system processes and immune responses, thereby enhancing colonic immunity and facilitating the absorption of crude fiber after degradation in Ding’an pigs.

Transcriptome differential expression analysis combined with weighted gene co-expression network analysis was used to explore the mechanism of coarse feeding tolerance in Ding’an pigs. One positively regulated gene, *LDHB*, was identified in Ding’an pigs. Three negatively regulated genes, namely *MLC1*, *LSG1*, and *ESM1*, were identified in Ding’an pigs. Lactate dehydrogenase B (*LDHB*) is an enzyme involved in glycolysis, catalyzing the conversion of lactate to pyruvate. Down-regulation of *LDHB* expression disrupts lactate-to-pyruvate conversion, leading to impaired mitochondrial ATP production and inhibition of oxidative phosphorylation. Overexpression of *LDHB* in mouse muscle enhances mitochondrial gene expression and respiratory capacity [59,60]. *MLC1*, also known as astrocyte membrane protein 1, played a crucial role in cerebral neurovascular development and physiology [61,62]. However, its physiological function in the colon remains unclear. *LSG1*, also known as the large 60S subunit nuclear GTPase 1, played a role in activating the 60S large subunit and ribosome biosynthesis. It was primarily located in the endoplasmic reticulum. *LSG1* and *EFl1* function sequentially in the final cytoplasmic maturation step of the 60S ribosomal subunit, facilitating the completion of the peptidyltransferase center (PTC) [63]. Endothelial cell-specific molecule 1 (*ESM1*), situated on chromosome 5q11.2, has been demonstrated to play a crucial role in regulating endothelial cell function. *ESM1* exhibited expression in vascular endothelial cells, distal renal tubular epithelial cells, bronchial tubes, and submucosal glands of the lung [64,65]. In this study, *LDHB* exhibited a positive correlation with the apparent digestibility of crude fiber, intramuscular fat, marbling score, and GLU and a negative correlation with shear force in Ding’an pigs. *MLC1*, *LSG1*, and *ESM1* exhibited negative correlations with the apparent digestibility of crude fiber and positive correlations with shear force in Ding’an pigs (Table 1). This indicated that up- or down-regulation of these genes might enhance the absorption of degraded crude fiber and contribute to the production of more tender meat in Ding’an pigs.

However, it is essential to acknowledge certain limitations in the study results. Genetic analyses were conducted with only four pigs per treatment group, which, while nearly adequate (though still low), might not suffice for determining meat quality traits. This limitation arises from the potential variability in meat quality traits among individuals or due to changes in slaughter conditions.

## 5. Conclusions

In this study, we established a database of phenotypes related to coarse feeding tolerance traits in Ding’an pigs, including growth performance, apparent digestibility of crude fiber, serum biochemical indices, carcass traits, and meat quality traits. Compared to DLY pigs, DA pigs exhibited higher crude fiber digestibility and superior meat quality but poorer growth performance and carcass traits. Increasing the level of crude fiber had less effect on growth performance, apparent digestibility of crude fiber, carcass traits, and meat quality traits in DA pigs, while DLY pigs became relatively worse. Through transcriptome differential expression analysis and WGCNA analysis, 65 and 482 central genes associated with coarse feeding tolerance in Ding’an pigs were identified, respectively. Comprehensive analyses identified four hub genes: *LDHB*, *MLC1*, *LSG1*, and *ESM1*, which were critical for coarse feeding tolerance in Ding’an pigs. Functional analyses of the differential candidate genes revealed their primary involvement in immune response signaling pathways. This study provides valuable data for further exploration of functional genes related to coarse feeding tolerance traits in Ding’an pigs.

## Figures and Tables

**Table 1:** Four hub genes of the turquoise module for the phenotypic significance of coarse feeding tolerance in Ding’an pigs.

GS	p.GS	MM	p.MM	Hub Gene	Trait
0.91	1.11 × 10^−6^	−0.97	1.46 × 10^−9^	*LDHB*	CF
0.75	0.00079	−0.97	1.46 × 10^−9^	*LDHB*	Intramuscular_fat
0.69	0.00274	−0.97	1.46 × 10^−9^	*LDHB*	Marbling_score
−0.90	1.81 × 10^−6^	−0.97	1.46 × 10^−9^	*LDHB*	Shear_force
0.80	0.00017	−0.97	1.46 × 10^−9^	*LDHB*	GLU
−0.80	0.00019	0.78	0.00034	*MLC1*	CF
0.71	0.00209	0.78	0.00034	*MLC1*	Shear_force
−0.65	0.00631	0.78	0.00038	*LSG1*	CF
0.86	0.00002	0.78	0.00038	*LSG1*	Shear_force
−0.62	0.00996	0.78	0.00038	*LSG1*	GLU
−0.61	0.01152	0.69	0.00294	*ESM1*	CF
0.73	0.00120	0.69	0.00294	*ESM1*	Shear_force

GS, Gene Significance; p.GS, the *p*-value for Gene Significance; MM, module membership; p.MM, the *p*-value for Module Membership.

## Data Availability

The original contributions presented in the study are included in the article and Supplementary Materials, further inquiries can be directed to the corresponding author. The data are not publicly available due to privacy restrictions.

## Supplementary Materials

The following supporting information can be downloaded at https://www.mdpi.com/article/10.3390/genes15050599/s1, Figure S1: (A) Scale-free network linear model of soft threshold β calculation results. (B) Network connectivity corresponding to different soft thresholds; Figure S2: Effect of pig breed and crude fiber level on Indicators. (A) pH _45 min_. (B) pH _24 h_. (C) Drip loss (%); Figure S3. Effect of pig breed and crude fiber level on Indicators. (A) ALB (g/L). (B) TC (mmol/L). (C) TG (mmol/L). (D) TP (g/L). Figure S4: Gene significance of each module–trait. Figure S5: Scatterplot of GS vs. MM different traits in the Turquoise module. cor indicates the correlation between GS and MM, and *p*-value indicates the significance of the correlation between GS and MM; Table S1: Maize-soybean meal ration composition and nutritional levels1. Table S2: Primers for real-time fluorescent quantitative PCR; Table S3: Differential Gene Expression for DAH vs. DAN, DLYH vs. DLYN, DAH vs. DLYH, DAN vs. DLYN; Table S4: 65 DEGs; Table S5: 482 DEGs in Turquoise module. Table S6: GO and KEGG functional enrichment of 482 DEGs in the Turquoise module.
