# Supplementary material for: Study on the Characteristics of Coarse Feeding Tolerance of Ding’an Pigs: Phenotypic and Candidate Genes Identification
Source: Genes (Basel). 2024 May 8;15(5):599. doi: 10.3390/genes15050599 (PMC11121715; doi:10.3390/genes15050599)
Supplement: Supplementary file 1 [file genes-15-00599-s001.zip › Supplementary Table S1. Maize-soybean meal ration composition and nutritional levels1..pdf]

Supplementary Table S1. Maize-soybean meal ration composition and nutritional levels[1].

Ingredients	Crude fiber5%	Crude fiber10%
Maize	49.40	32.70
Alfalfa grass meal	2.70	16.90
Bran	13.10	13.10
Soybean meal	18.00	18.00
Rice bran	13.00	13.00
Soybean oil	1.00	3.50
Stone meal	1.40	1.40
Salt	0.30	0.30
Lysine	0.10	0.10
Premix	1.00	1.00
Total	100.00	100.00
Nutrient level		
Digestive energy (DE)	13.00	12.89
Crude protein (CP)	16.01	16.30
Lysine	0.85	0.84
Methionine + cystine	0.44	0.43
Tryptophan	0.14	0.14
Threonine	0.54	0.53
Crude fiber	5.00	10.00
Calcium	0.64	0.65
Total phosphorus	0.55	0.54

[1] Digestive energy was calculated, and the rest was measured. The Maize-soybean meal ration were quoted from Pengxiang Xue's article
