# Supplementary figures and images for: Study on the Characteristics of Coarse Feeding Tolerance of Ding’an Pigs: Phenotypic and Candidate Genes Identification
Source: Genes (Basel). 2024 May 8;15(5):599. doi: 10.3390/genes15050599 (PMC11121715; doi:10.3390/genes15050599)

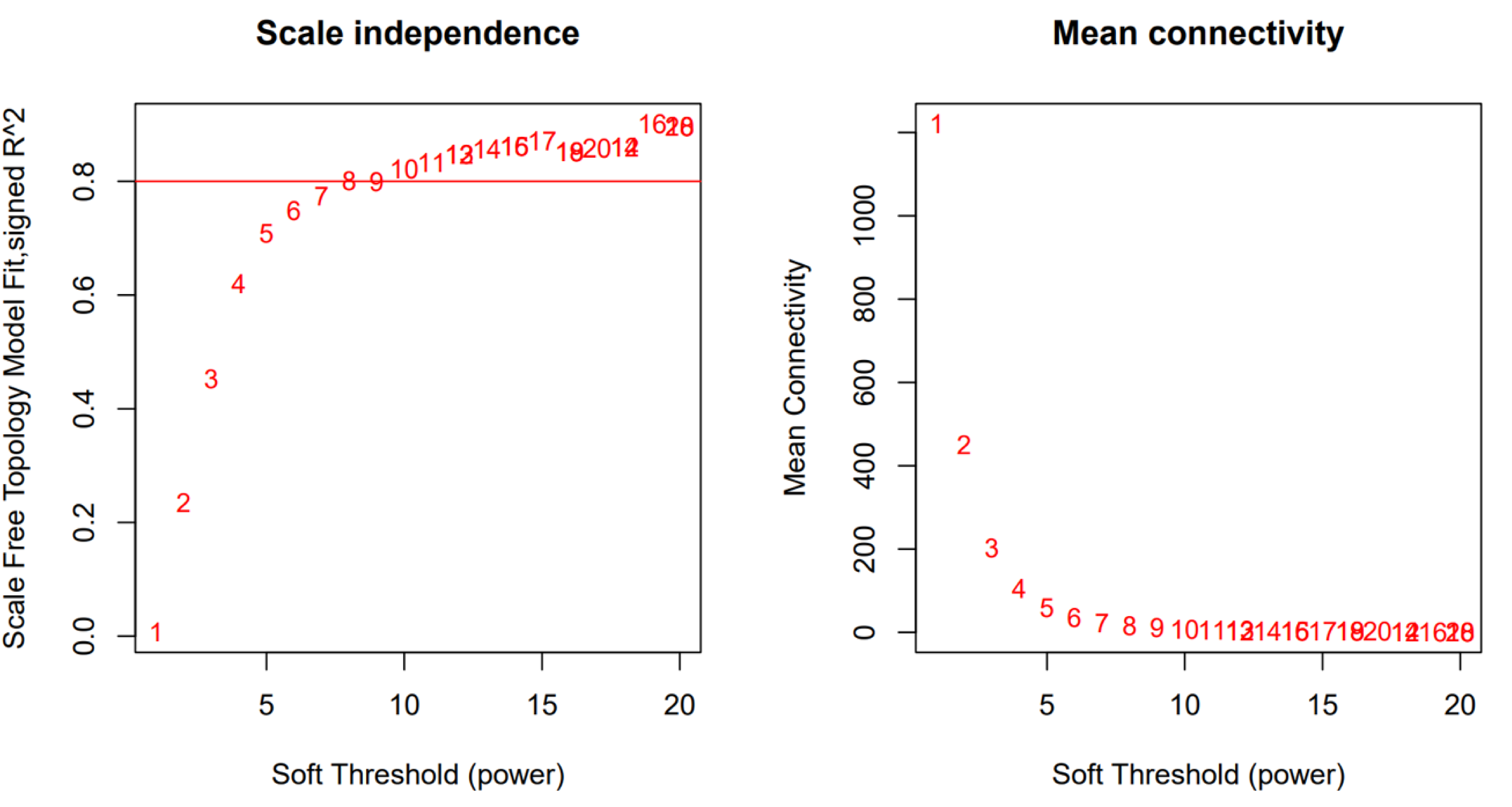

Supplement: Supplementary file 1 [file genes-15-00599-s001.zip › Supplementary FigureS1..tif]

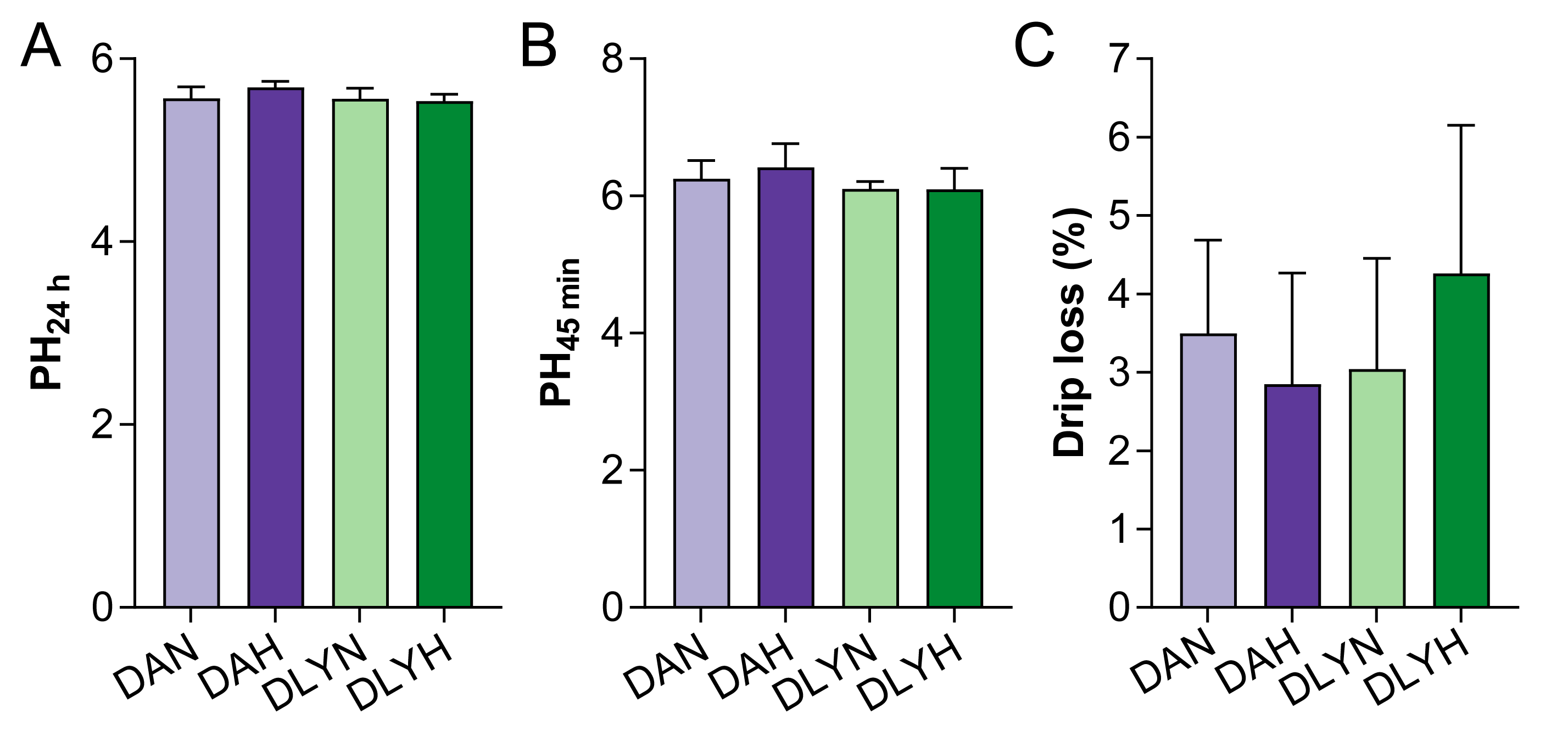

Supplement: Supplementary file 1 [file genes-15-00599-s001.zip › Supplementary FigureS2..tif]

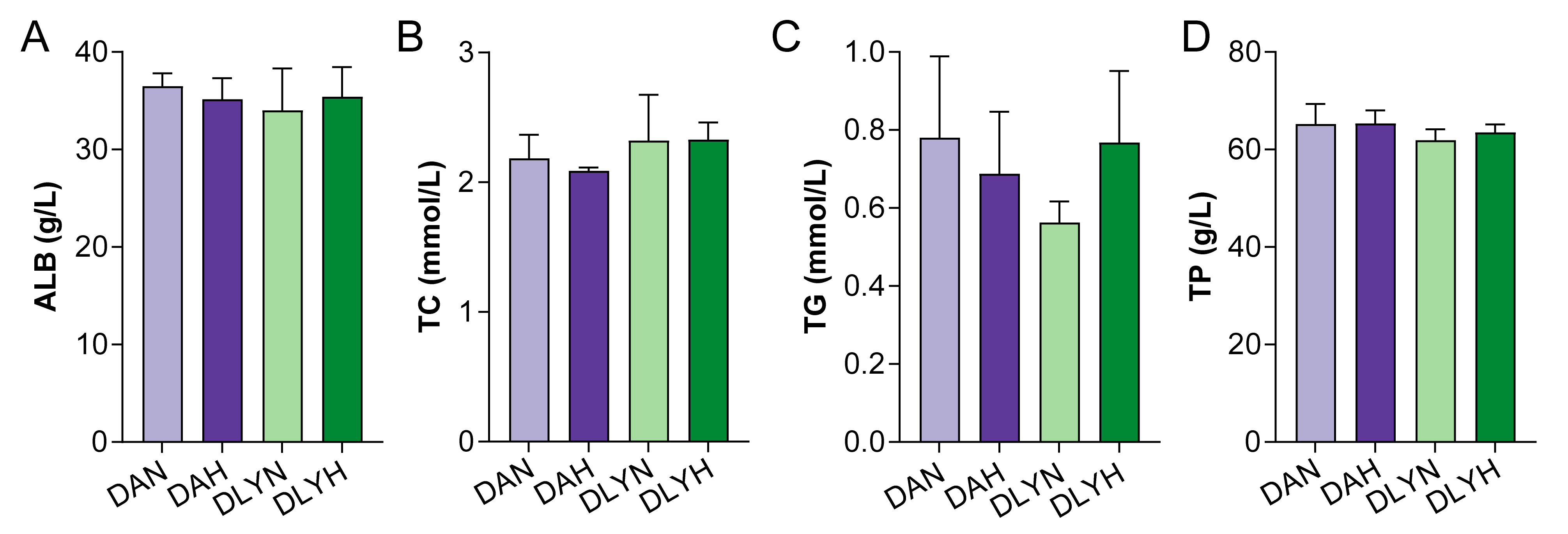

Supplement: Supplementary file 1 [file genes-15-00599-s001.zip › Supplementary FigureS3..tif]

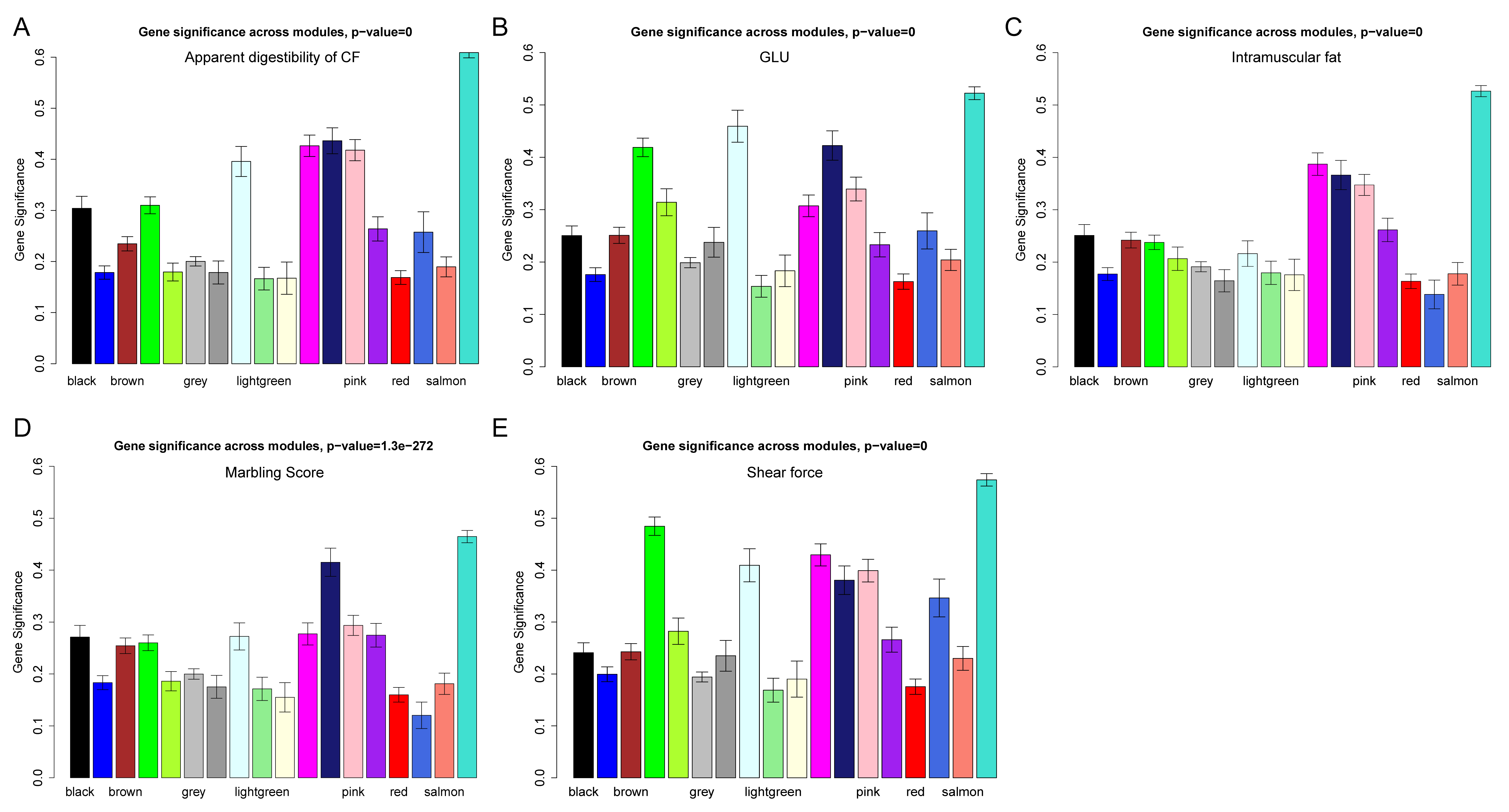

Supplement: Supplementary file 1 [file genes-15-00599-s001.zip › Supplementary FigureS4..tif]
